# Risk Stratification in Patients With Follicular Neoplasm on Cytology: Use of Quantitative Characteristics and Sonographic Patterns

**DOI:** 10.3389/fendo.2021.614630

**Published:** 2021-04-30

**Authors:** Ming-Hsun Wu, Kuen-Yuan Chen, Min-Shu Hsieh, Argon Chen, Chiung-Nien Chen

**Affiliations:** ^1^ Department of Surgery, National Taiwan University Hospital, Taipei, Taiwan; ^2^ Department of Pathology, National Taiwan University Hospital, Taipei, Taiwan; ^3^ Graduate Institute of Industrial Engineering, National Taiwan University, Taipei, Taiwan

**Keywords:** follicular neoplasm, ultrasonography, thyroid gland, thyroid cancer (TC), follicular thyroid carcinoma (FTC)

## Abstract

**Objectives:**

Differentiating thyroid nodules with a cytological diagnosis of follicular neoplasm remains an issue. The goal of this study was to determine whether ultrasonographic (US) findings obtained preoperatively from the computer-aided detection (CAD) system are sufficient to further stratify the risk of malignancy for this diagnostic cytological category.

**Methods:**

From September 2016 to September 2018 in our hospital, patients diagnosed with Bethesda category IV (follicular neoplasm or suspicion of follicular neoplasm) thyroid nodules and underwent surgical excisions were include in the study. Quantification and analysis of tumor features were performed using CAD software. The US findings of the region of interest, including index of composition, margin, echogenicity, texture, echogenic dots indicative of calcifications, tall and wide orientation, and margin were calculated into computerized values. The nodules were further classified into American Thyroid Association (ATA) and American College of Radiology Thyroid Imaging Reporting & Data System (TI-RADS) categories.

**Results:**

92 (10.1%) of 913 patients were diagnosed with Bethesda category IV thyroid nodules. In 65 patients, the histological type of the nodule was identified. The quantitative features between patients with benign and malignant conditions differed significantly. The presence of heterogeneous echotexture, blurred margins, or irregular margins was shown to have the highest diagnostic value. The risks of malignancy for nodules classified as having very low to intermediate suspicion ATA, non-ATA, and high suspicion ATA patterns were 9%, 35.7%, and 51.7%, respectively. Meanwhile, the risks of malignancy were 12.5%, 26.1%, and 53.8% for nodules classified as TIRADS 3, 4, and 5, respectively. When compared to human observers, among whom poor agreement was noticeable, the CAD software has shown a higher average accuracy.

**Conclusions:**

For patients with nodules diagnosed as Bethesda category IV, the software-based characterizations of US features, along with the associated ATA patterns and TIRADS system, were shown helpful in the risk stratification of malignancy.

## Introduction

Thyroid nodular disease is one of the most common endocrine disorders. The preoperative diagnosis of thyroid nodule is commonly based on fine-needle aspiration cytology (FNAC) results (1). However, follicular cell-derived thyroid nodules have overlapping cytomorphological features (2–4); thus, 15%-30% of aspirations are indeterminate (5). The Bethesda system of thyroid cytopathology defines follicular neoplasm (FN) or suspicion of follicular neoplasm (SFN) as Bethesda category IV, with the goal of increasing the probability of detecting follicular thyroid cancers (6). It reports a 10% to 40% association with malignancy risk, and diagnostic thyroidectomy is often recommended for making a diagnosis (7–9). Moreover, up to 85% of thyroidectomy procedures are performed for benign lesions in this category (10). The ATA guidelines suggest consider using assessment methods for molecular markers, such as mutational testing using next-generation sequencing or special immunohistochemical staining, rather than the operative approach for diagnosis. However, these methods are expensive and currently are not widely available.

High-resolution ultrasonography (US) is the initial imaging technique used to evaluate the gross morphologic characteristics of thyroid nodules. Most studies of the US features of thyroid nodules have focused on how to select patients for further cytological examinations, while few studies have reported the predictive value of US for malignant nodules in Bethesda category IV. In addition, the results of these studies were not conclusive (1, 11, 12). Our previous study showed that certain gray-scale US features may help differentiate pathologically confirmed follicular thyroid cancer (FTC) from follicular adenoma (FA) (13). However, tumors with Bethesda category IV cytology do not always result in a histologic diagnosis of FTC and FA, and it remains uncertain whether they can be used to differentiate this category preoperatively. In addition, algorithms that can identify patients at high risk of malignancy may allow surgeons to perform a single definitive operation (usually lobectomy or total thyroidectomy if needed), thereby saving time, reducing patient morbidity, and reducing costs.

The major limitations of thyroid US are inter-observer and intra-observer variability (14–19). Computerized quantification methods that can characterize the sonographic features to make the diagnosis more objective are available, and have shown acceptable agreement with experienced clinicians (20–26). A computer-aided detection (CAD) system (K180006) had been developed based on these methods and validated following the guidelines of the United States Food and Drug Administration (FDA) for clinical use.

Because further differentiation of Bethesda category IV lesions is challenging, we aimed to examine whether the information provided by commercial CAD to surgeons is sufficient to predict thyroid cancer. Moreover, we examined the usefulness of the current ATA and TIRADS systems in surgical planning for these patients.

## Materials and Methods

### Participants

The institutional review board of the National Taiwan University Hospital approved the data collection and analyses in this study. Based on thyroid FNACs performed during the study period from September 2016 to September 2018 in the National Taiwan University Hospital, 92 (10.1%) of 913 patients were diagnosed with Bethesda category IV thyroid nodules. Because molecular testing is not widely available in Taiwan, physicians recommended surgical excision to these patients for the removal and definitive diagnosis of an FN/SFN thyroid nodule. Sixty-five patients who underwent thyroidectomy were recruited in this study. The diagnoses were based on the histopathological findings of surgical specimens that were assessed by pathologists. The other 27 patients refused to undergo an operation.

### Equipment and Ultrasonographic Procedures

All sonograms were performed using commercial ultrasonography devices with multi-frequency (4-12 megahertz) linear probes set to the highest frequency available, including HDI 5000 (Philips Medical Systems, Bothell, WA, the USA), Voluson 730 (GE Medical Systems, Milwaukee, WI, the USA), and ALOKA ProSound 2 (Hitachi Medical Systems Europe, Steinhausen, Switzerland). The procedure was performed while the participant was in a supine position with the neck hyperextended. Images were captured using the maximum nodule diameter. Image analysis was conducted offline using the DICOM format of images on a separate computer. Quantification of tumor features was performed using a commercial software (AmCAD-UT, AmCad BioMed, Taiwan).

Briefly, the operators provided the four endpoints of the axes on the thyroid nodule margins. The CAD software was used to calculate the contour of the mass, to distinguish it from normal thyroid tissues. In the image analysis session, the US findings of the region of interest (ROI), including index of composition (CoI), index of margin (MI), index of echogenicity (EI), index of texture (TI), index of echogenic dots indicative of calcifications (CaI), index of tall and wide orientation (TWI), and index of margin irregularity (MII), were calculated into computerized values.

CoI calculated the percentage of the cystic component consisting of the anechoic pixels. The anechoic pixels were those pixels with the gray-scale intensity readings below a certain pre-defined threshold inside the ROI (20). CaI was the proportion of the echogenic foci in the solid component of ROI. The solid component was defined to be the area of the ROI excluding the cystic area. Echogenic foci were then represented by those hyperechoic pixels with the gray-scale intensity readings higher than a pre-defined threshold (20). EI was the difference between the average gray-scale intensities of the ROI area and the surrounding reference area outside the ROI (21). TI was to compute the texture heterogeneity of the solid component by calculating the texture variation among small areas within the ROI (22). The higher TI value indicated a greater composition difference of the nodule tissue. TWI calculated the height to width ratio with the nodule height measured along the sound-beam direction and the nodule width in the direction perpendicular to the height as defined by ACR. MI was an indicator of the ill-defined margin that calculated the proportion of the indistinctive border between the nodule and the thyroid parenchyma (27). MII was computed to describe how irregular the nodule margin was by comparing the radius variation of the ROI margin to that of a smooth margin defined by the nodule long and short axes and a corresponding Bezier curve.

These index values were then quantified to represent features that described the thyroid nodule, including factors such as solid or cystic; well-defined or ill-defined; hyperechoic/isoechoic or hypoechoic/markedly hypoechoic; heterogeneous or homogeneous; presence of calcification; presence of taller-than-wide orientation; and presence of irregular margin (20–23).

CAD software was used to classify nodules according to the 2015 ATA sonographic patterns (CAD-ATA), using the referral description of the sonographic features (28). Scenarios not described in the 2015 ATA classification were classified into a separate category referred to as non-ATA patterns. These scenarios included heterogenous nodules with or without other suspicious features, and iso- or hyperechoic nodules with at least one suspicious feature.

CAD software was also utilized to calculate the total risk score of each thyroid nodule by summing the scores of each US feature and classifying them into TIRADS categories (CAD-TIRAD) (29).

Recorded images of thyroid ultrasound were also independently reviewed by three human observers (MH Wu, KY Chen, and MF Wang), all with more than 10 years’ experience in thyroid ultrasound. They interpreted each nodule with 2015 ATA sonographic patterns (Observer-ATA) and TIRADS categories (Observer-TIRAD). If there are discordance in their results, the final interpretation was determined by consensus of two or more observers. When there was no consensus through the independent assessment, the image was reviewed jointly to reach a consensus.

The malignancy rate of each of the ATA and TIRADS category was calculated based on the final histological outcomes of the nodules.

### Pathological Evaluation

The decision to biopsy thyroid nodules in our institute was based on 2015 ATA recommendations according to sonographic patterns and sizes. Cytological diagnoses were made using the Bethesda System for Reporting Thyroid Cytopathology by board-certified cytologists, and were retrieved from the cytology reports (6, 30). In our thyroid FNAC practice, cases with inconsistent diagnoses were reviewed and agreement was achieved by discussion.

### Statistical Analysis

Data were summarized using descriptive statistics. Categorical variables were presented as numbers and percentages, while continuous variables were presented as means and standard deviations or mediums and ranges for those tested significantly not conforming to normal distributions. Data on patient characteristics were analyzed with Student’s *t*-test or analysis of variance for continuous variables, Mann-Whitney test for non-normal continuous variables and chi-square test or Fisher’s exact test for categorical variables. Diagnostic performance was estimated by Positive Predictive Value (PPV), Negative Predictive Value (NPV), Sensitivity, and Specificity, all calculated with 95% Confidence Intervals (95% CI). Statistical analyses were performed using SAS 9.4 (Cary, NC, USA). All p-values were two-sided, and the significance level was set at 5%.

## Results

Based on thyroid FNACs performed during the study period, 92 (10.1%) of 913 patients were diagnosed with Bethesda category IV thyroid nodules. Among them, 27 patients refused to undergo an operation. There were no significant age, sex, and nodule size differences in those with and without surgeries in this study. In total, 65 patients who underwent surgery were included for further analysis. Of these, 22 had cancer in the thyroid nodule, based on histopathological examination, i.e., a malignancy rate of 33.8% (22/65).

he demographic characteristics of the 65 patients with 65 thyroid nodules are summarized in **Table 1**. Histopathology results revealed that 19 nodules were nodular goiter; 24 were FA; 15 were papillary thyroid cancer (PTC), including four follicular variant subtypes; and 7 were FTC. None of the patients in this study had Noninvasive follicular thyroid neoplasm with papillary-like nuclear features (NIFTP). The average width, height, and length of the nodules were 2.2 ± 1.3, 1.6 ± 0.8, and 3.3 ± 1.5 cm, respectively.

**Table 1:** Demographic characteristics of patients diagnosed with follicular neoplasm on cytology.

Characteristics	Number (%) or mean ± SD (range)
Gender	
Male	10 (15.4)
Female	55 (84.6)
Age	46.8 ± 14.1 (18–73)
Histological diagnosis	
Nodular goiter	19(29.2)
Follicular/Hurthle cell adenoma	24(36.9)
Papillary carcinoma	15(23.1)
Follicular/Hurthle cell carcinoma	7(10.8)
Location of nodules	
Left	22 (33.8)
Right	41 (63.1)
Isthmus	2 (3.1)
Size of nodules (cm)	
Length	2.38 ± 1.28 (0.72–7.28)
Width	2.18 ± 1.26 (0.56–7.19)
Height	1.62 ± 0.82 (0.35–4.72)

**Figure 1:**
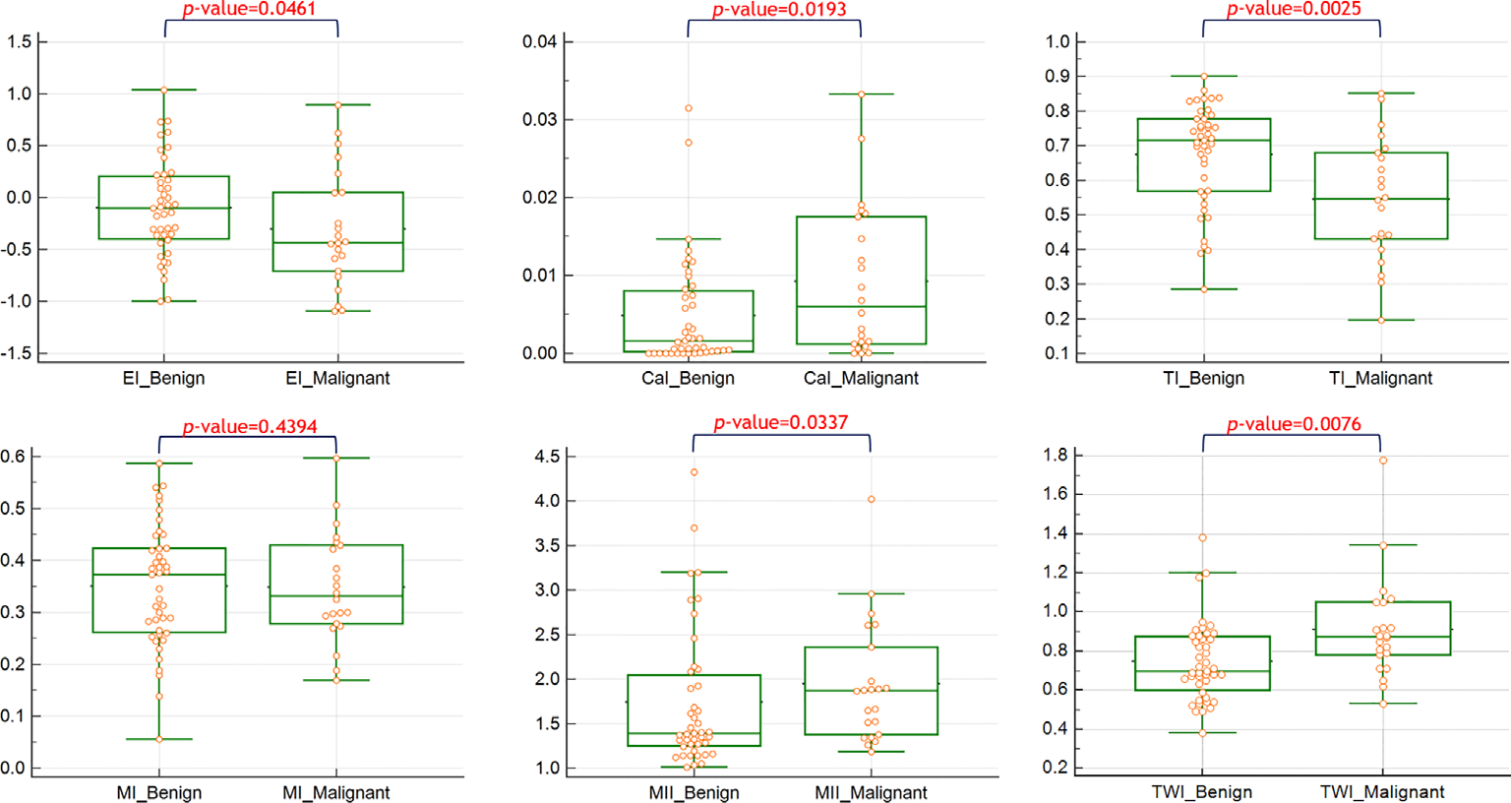
The quantitative values of the features between benign and malignant nodules.

The quantitative values of the features between benign and malignant nodules are shown in **Figure 1**. Significant differences were observed in the feature indices of CaI, EI, TI, TWI, and MII.

The demographic and sonographic characteristics of patients with benign and malignant nodules are presented in **Table 2**. No significant differences were observed in terms of age and sex distribution (p = 0.64 and 0.076, respectively). A higher number of malignant tumors had heterogeneous echotexture (15/22, 68.2% versus 14/43, 32.6%, p = 0.0067) and taller-than-wide orientation (6/22, 27.3% versus 3/43, 7.0%, p = 0.0261).

**Table 2:** Comparison of the demographic characteristics of the participants and sonographic characteristics between thyroid nodules with benign and malignant histology.

	Benign (n = 43)	Malignant (n = 22)	*p* value
Gender			0.076^F^
Male/female	4/39	6/16	
Age	47 ± 13.9, 18–73	46.4 ± 14.7, 23–72	0.64^t^
Location of nodule			0.63^F^
Left/right/isthmus	16/26/1	6/15/1	
Size of the nodule (cm)			
Length	2.3 (0.92–7.28)	1.55 (0.72–5.12)	0.03*
Width	2.07 (0.64–7.19)	1.41 (0.56–5.20)	0.05*
Height	1.54 (0.35–3.87)	1.2 (0.45–4.72)	0.27*
Sonographic characteristics			
Taller-than-wide Morphology	3 (7.0%)	6 (27.3%)	**0.0261** ^†^
Component			0.17^F^
Solid/mixed	30/13 (69.8%)	11/11 (50.0%)	
Calcification			0.86^F^
Macro/eggshell/micro	2/4/13	1/2/12	
Hypo-echogenicity	20 (46.5%)	15 (68.2%)	0.10^†^
Heterogeneous echotexture	14 (32.6%)	15 (68.2%)	**0.0067** ^†^
Blurred margins	11 (25.6%)	9 (40.9%)	0.2087^†^
Irregular margins	11 (25.6%)	9 (40.9%)	0.2087^†^

^F^These variable were compared using the Fisher’s exact test.

^†^These variable were compared using the chi-square test.

^t^These variable were compared using the Student’s t-test.

*These variable were compared using the Mann-Whitney U test.

Bold denotes statistically significant results.

To learn the preoperative diagnostic value of the US features determined by CAD in differentiating benign from malignant nodules, we calculated the positive and negative predictive values and the sensitivity, specificity, and average accuracy (average sensitivity and specificity), as shown in **Table 3**. Using a combination of three features, namely, heterogeneous echotexture, irregular margin, and blurred margin, was found to have the highest average accuracy (0.735). 47% of benign Bethesda category IV nodules were with none of these features and 100% of malignant nodules were accurately diagnosed by having at least one of the features present.

**Table 3:** Sonographic features used in differentiating benign from malignant conditions in 65 patients with follicular neoplasms.

	PPV (95% CI)	NPV (95% CI)	Sensitivity (95% CI)	Specificity (95% CI)	Average*
Sonographic characteristics					
Hypo-echogenicity	0.44 (33.7-55.1)	0.77 (63.8-87.0)	0.68 (45.1-86.1)	0.56 (39.9-70.9)	0.62
Calcification	0.48 (33.8-62.6)	0.75 (64.6-83.2)	0.55 (32.2-75.6)	0.70 (53.9-82.8)	0.625
Heterogeneous echotexture	0.52 (39.0-64.2)	0.81 (68.5-88.8)	0.68 (45.1-86.1)	0.67 (51.5-80.9)	0.675
Blurred margin	0.45 (28.6-62.6)	0.71 (62.5-78.4)	0.41 (20.7-63.6)	0.74 (58.8-86.5)	0.575
Irregular margin	0.45 (28.6-62.6)	0.71 (62.5-78.4)	0.41 (20.7-63.6)	0.74 (58.8-86.5)	0.575
Taller-than-wide morphology	0.67 (35.6-87.9)	0.71 (65.6-76.6)	0.27 (10.7-50.2)	0.93 (80.9-98.5)	0.6
Combination of heterogeneous, irregular margin, and blurred margin	0.49 (42.0-55.8)	1.0 (-)	1.0 (84.6-100.0)	0.47 (31.2-62.3)	0.735

*Average sensitivity and specificity.

The nodules were classified into the 2015 ATA sonographic patterns and TIRADS categories based on the sonographic characteristics by both CAD and human observers. For CAD, none of the nodules had an ATA benign pattern. Twenty-two nodules were classified as very low to intermediate suspicion patterns, 29 as high suspicion patterns, and 14 as non-ATA patterns. The risk of finding malignant nodules with very low to intermediate suspicion patterns, non-ATA patterns, and high suspicion patterns were 9%, 35.7%, and 51.7%, respectively. None of the nodules were classified as TIRADS 1 and 2 in our study. Meanwhile, 16, 23, and 26 nodules were classified as TIRADS 3, 4, and 5, respectively. The risk of developing malignant nodules in each TIRADS category increased with increasing TIRADS score: TIRADS 3, 12.5%; TIRADS 4, 26.1%; and TIRADS 5, 53.8% (**Table 4**).

**Table 4:** Malignancy rate based on the ATA and TIRADS category in 65 patients diagnosed with follicular neoplasm on cytology.

	CAD	No. of cases (n=65)	Benign(n=43)	Malignant (n=22)	Risk of Malignancy, %	Observers	No of cases (n=65)	Benign(n=43)	Malignant (n=22)	Risk of Malignancy, %
ATA category	Benign to Intermediate	22	20	2	9.1	Benign to Intermediate	18	15	3	16.7
	Non-ATA	14	9	5	35.7	Non-ATA	3	3	0	0
	High	29	14	15	51.7	High	44	25	19	43.2
TIRADS category	2~3	16	14	2	12.5	2~3	4	3	1	25.0
	4	23	17	6	26.1	4	33	26	7	21.2
	5	26	12	14	53.8	5	28	14	14	50.0

For human observers, the intraclass correlation coefficients (ICC) for absolute agreements of the distributions of the 2015 ATA sonographic patterns and TIRADS categories among observers were poor to fair with r = 0.24 [CI 0.09–0.40] and r=0.44[CI 0.29-0.58], respectively (**Supplementary Table 1**). The risk of finding malignant nodules by observer-consensus with benign to intermediate suspicion patterns, non-ATA patterns, and high suspicion patterns were 16.7%, 0%, and 43.2%, respectively. The malignant rate by observer-consensus was 25% for TIRADS 1-3, 21.2% for TIRADS 4, and 50% TIRADS 5 (**Table 4**).

The diagnostic values of the ATA/TIRADS determined by CAD and observers are shown in **Table 5**. The CAD software appeared to have higher average accuracies than the observers (0.675/0.68 and 0.64/0.655, respectively).

**Table 5:** Comparison between CAD and Observers.

		**PPV** **(95% CI)**	**NPV** **(95% CI)**	**Sensitivity** **(95% CI)**	**Specificity** **(95% CI)**	Average*
CAD Performance	ATA category of high suspicion	0.52 (39.0-64.2)	0.81 (68.5-88.8)	0.68 (45.1-86.1)	0.67 (51.5-80.9)	0.675
	TIRADS category of highly suspicious	0.54 (39.6-67.5)	0.80 (68.4-87.4)	0.64 (40.7-82.8)	0.72 (56.3-84.7)	0.68
Observers Performance	ATA category of high suspicion	0.43 (35.9-50.7)	0.86 (66.4-94.8)	0.86 (65.1-97.1)	0.42 (27.0-57.9)	0.64
	TIRADS category of highly suspicious	0.50 (37.0-63.0)	0.78 (66.8-86.7)	0.64 (40.7-82.8)	0.67 (51.5-80.9)	0.655

*Average sensitivity and specificity.

## Discussion

Follicular thyroid neoplasms have numerous clinical and cytological characteristics, and it is difficult to achieve a precise diagnosis preoperatively. The use of clinical manifestations, imaging study results, and even findings of fine-needle aspiration/biopsy of thyroid nodules is not optimal. Resected surgical specimens are important in obtaining an accurate diagnosis of thyroid follicular neoplasms. The ATA guidelines recommend removal and definitive diagnosis of an FN/SFN thyroid nodule, if molecular testing is not performed or inconclusive. However, only 10%-40% of nodules in this category are found to be malignant based on histopathology examination (28, 31). The management of these thyroid nodules, prevention of unnecessary thyroidectomy, and determination of the extent of resection are extremely important in this clinical setting (32). The fact that most patients in the current study have benign diseases based on postoperative histology examination, which is consistent with previous studies (28, 31), justifies the effort to improve the selection of surgery candidates.

Studies have shown that the clinical predictive factors for malignancy in Bethesda category IV nodules include sex and age at diagnosis (5, 33, 34). In this study, we found no difference in terms of sex and age between patients with benign and malignant nodules. Some studies have shown that a larger nodule size is indicative of malignancy. However, the current study and other studies have shown that nodule size is not a predictive factor of malignancy in patients with Bethesda category IV nodules (1, 35–37). Moreover, nodules that were found to be Bethesda category IV by FNAC and were subsequently surgically resected had been previously reported to be larger than those that were not resected (38). However, no significant size differences between nodules with and without surgeries were observed in this study (data not shown). Regarding the pathology distribution, 22 of 62 (33.8%) Bethesda IV nodules were cancers with 15 papillary thyroid cancers being the most common type of cancers and 7 follicular and Hurthle cell carcinomas accounting for about 10% of cases. The distribution appears to be consistent with previous studies (4, 39, 40), where 13~22% of Bethesda IV nodules were PTC or follicular variant PTC and about 9% were follicular or Hurthle cell carcinoma. Although a significant proportion of patients with malignant nodules presented with papillary thyroid cancers, four of them had follicular variants of papillary thyroid cancers.

US is widely used for the screening of thyroid nodules. The presence of US features indicates a malignancy potential for thyroid nodules (41–44). Based on these findings, the nodule can be categorized, and decisions can be made as to whether FNAC or follow-up is required (5, 11, 29, 38). Studies about post-FNAC stratification are limited, and few studies have explored the US features of follicular neoplasm (45, 46). In the current study, the presence of heterogenous echotexture and taller‐than‐wide features was higher in malignant than benign follicular neoplasms. In the past, heterogeneity echotexture was found to be not as important as other sonographic features, and was not listed in the current ATA and TIRDAS category system. However, in the current study and in our previous work, heterogeneous echotexture is associated with an increased risk of malignancy, particularly for follicular neoplasms (13, 22). Studies have shown that a taller-than-wide feature is extremely specific for malignant thyroid nodules. However, information about such features in follicular neoplasms is limited (28, 43, 47, 48).

Because US is a relatively subjective diagnostic method, observers may have different opinions when describing and interpreting lesions (14, 49), leading to poor reliability for some features (14, 16, 17, 19, 50–56). Several studies have shown that inconsistencies in the interpretation of ultrasonographic features occur in up to 70% of cases (17, 19, 56). This low reproducibility, that may cause uncertainty in clinical management of thyroid nodules, emphasizes the need for an objective quantification method, such as a software device to computerize these features (54). The sensitivity and specificity of US findings vary in the literature (44, 57). The diagnostic performance of thyroid US in Bethesda category IV nodules has a lower sensitivity (50%) and positive predictive value (PPV) (50%) than that in other Bethesda categories. Thus, the importance of the current US morphological guidelines for this category is limited (58). The CAD software system, as used in this study, can reduce the difference in feature interpretation, and the reader experiences a gap while increasing reading accuracy, which may be used as an accompaniment in imaging diagnosis (20–23, 59).

We showed a difference in the quantitative index of echogenicity, calcification, echotexture, margin and tall-wide orientation in a malignant tumor with Bethesda category IV, which is different from a ‘yes’ or ‘no’ binary feature presentation. Quantitative indices provide detailed comparisons and can establish more precise predictive models in future studies.

The role of sonographic features or patterns should not only be used in selecting nodules that must be biopsied, but also in determining the management after cytological diagnosis (60). Recently, it was determined that the rate of malignancy in these types of nodules can be stratified according to sonographic patterns (60, 61). In this study, the features were used to classify using the ATA and ACR TIRAD, which are the most popular category systems to differentiate the risk of malignancy. This showed that both systems can help stratify those who may benefit from or who should have a pathological diagnosis. However, this study also showed that a relatively high percentage (21.5%, 14 of 65) of tumors had non-ATA patterns, and that there was a large gap in the malignancy rate (9.1%–51.7%) from ATA very low, low, intermediate to ATA high suspicion. In this study, the ACR TIRADS system included more feature information, and the malignant rate increased (12.5%-53.8%) with advancing CAD TIRADS scores as indicated in **Table 4**. This suggests that this approach may result in better stratification for Bethesda category IV tumors. Compared with the risk of malignancy of Bethesda category IV, which is highly uncertain with a range from 10% to 40%, the additional risk stratification by CAD TIRADS will further help the surgery decision. Once more studies with greater sample sizes are conducted to confirm the findings, nodules categorized with Bethesda IV and TIRADS 3 or below would be considered low risk of malignancy and may undergo continuous follow-up. Bethesda IV nodules categorized with TIRADS 4 would be considered moderate risk of malignancy and lobectomy could be suggested. A high risk of malignancy is seen in nodules categorized with Bethesda IV and TIRADS 5, and at least lobectomy or total thyroidectomy for larger nodules is suggested. Numerous variations in TIRADS have been proposed, and some of them included additional US features, such as elastography and vascularity criteria (47). The ACR TIRADS used in our study is simple, convenient, and accurate and can be used to stratify the risk of malignancy. Furthermore, we showed that 30.8% of follicular neoplasms do not present features such as heterogeneity, blurred margin, and irregular margin; their malignancy rate is 0%. These patients could be spared from surgery and monitoring. However, further large-scale studies would need to confirm the findings of the current study.

However, we found in the study that there were differences between the human observers and the CAD in ATA/TIRAD interpretation. Even among the human observers, the consistence of interpretation was also shown to be poor or fair. Thus, further large-scale studies must be conducted to confirm the findings of the current study.

This study had several limitations: first, we only included patients who underwent surgery and therefore potential selection bias may have affected the study results. Nevertheless, this bias was inevitable because histopathology was necessary to provide a reliable diagnosis in patients with Bethesda category IV nodules. Second, in order to acquire dicom-format images for software analysis, all patients enrolled in this study were arranged for additional ultrasound scans. Therefore, collecting an extremely large number of cases within a short period of time is impossible. Studies with a larger number of cases should be conducted when the CAD software can be applied more widely to routine clinical examination with a standard workflow. Third, the applicability of this study should be limited to those nodules pre-selected for FNAC based on ATA guidelines and diagnosed as Bethesda IV category. Fourth, instead of real-time images during ultrasonography, only static images selected and recorded by the US examiners were analyzed in this study. Thus, the advantage of dynamic US examination was not considered in the current study. In addition, because of the nature of this study, elastography or vascularization was not available in our workflow.

## Conclusion

For patients with nodules pre-selected for FNAC based on ATA guidelines and diagnosed as Bethesda category IV, the software-based characterizations of US features, along with the associated ATA patterns and TIRADS system, were shown helpful in the risk stratification of malignancy.

## Data Availability Statement

The raw data supporting the conclusions of this article will be made available by the authors, without undue reservation.

## Ethics Statement

The studies involving human participants were reviewed and approved by the institutional review board of the National Taiwan University Hospital approved the data collection and analyses in this study. The patients/participants provided their written informed consent to participate in this study.

## Author Contributions

M-HW, C-NC, K-YC, AC and M-SH proposed the study. M-HW, C-NC, K-YC and AC performed research, analyzed the data and wrote the first draft. M-SH collected the data. M-HW, C-NC, K-YC and AC analyzed the data. All authors contributed to the design and interpretation of the study and to further drafts. All authors contributed to the article and approved the submitted version.

## Funding

The AmCad BioMed Corporation, Taipei, Taiwan, sponsored this study in terms of technical assistance and financial support.

## Conflict of Interest

M-HW, K-YC, AC and C-NC received research grant support from AmCad BioMed.

The remaining author declares that the research was conducted in the absence of any commercial or financial relationships that could be construed as a potential confict of interest.
