# Supplementary material for: Risk Stratification in Patients With Follicular Neoplasm on Cytology: Use of Quantitative Characteristics and Sonographic Patterns
Source: Front Endocrinol (Lausanne). 2021 Apr 30;12:614630. doi: 10.3389/fendo.2021.614630 (PMC8120278; doi:10.3389/fendo.2021.614630)
Supplement: Supplementary file 1 [file Table_1.docx]

Supplementary Table 1. Differences in Frequency of ATA and TIRADS between Observers

|  | ATA category | Benign to Intermediate | Non-ATA | High Suspicious | TIRADS category | 1~3 | 4 | 5 |
| --- | --- | --- | --- | --- | --- | --- | --- | --- |
| Frequency | Observer 1 | 9 (13.8) | 4 (6.2) | 52 (80.0) | Observer 1 | 4 (6.2) | 27 (41.5) | 34 (52.3) |
|  | Observer 2 | 16 (24.6) | 6 (9.2) | 43 (66.2) | Observer 2 | 4 (6.2) | 28 (43.1) | 33 (50.8) |
|  | Observer 3 | 25 (38.5) | 2 (3.0) | 38 (58.5) | Observer 3 | 7 (10.8) | 36 (55.4) | 22 (33.8) |
|  | ICC | 0.24 [0.09-0.40] | | | ICC | 0.44 [0.29-0.58] | | |

ICC: intraclass correlation coefﬁcient with conﬁdence interval
